# Area under the expiratory flow-volume curve: predicted values by artificial neural networks

**DOI:** 10.1038/s41598-020-73925-0

**Published:** 2020-10-06

**Authors:** Octavian C. Ioachimescu, James K. Stoller, Francisco Garcia-Rio

**Affiliations:** 1 Division of Pulmonary, Allergy, Critical Care and Sleep Medicine, School of Medicine, Emory University, Atlanta VA Sleep Medicine Center, 250 N Arcadia Ave, Decatur, GA 30030 USA; 2 Jean Wall Bennett Professor of Medicine, Chair-Education Institute, Cleveland Clinic, 9500 Euclid Ave, Cleveland, OH USA; 3 Servicio de Neumología, Hospital Universitario La Paz, IdiPAZ-Departamento de Medicina, Universidad Autónoma de Madrid-Centro de Investigación Biomédica en Red en Enfermedades Respiratorias (CIBERES), Madrid, Spain

**Keywords:** Respiration, Respiratory tract diseases

## Abstract

Area under expiratory flow-volume curve (AEX) has been proposed recently to be a useful spirometric tool for assessing ventilatory patterns and impairment severity. We derive here normative reference values for AEX, based on age, gender, race, height and weight, and by using artificial neural network (ANN) algorithms. We analyzed 3567 normal spirometry tests with available AEX values, performed on subjects from two countries (United States and Spain). Regular linear or optimized regression and ANN models were built using traditional predictors of lung function. The ANN-based models outperformed the de novo regression-based equations for AEX_predicted_ and AEX z scores using race, gender, age, height and weight as predictor factors. We compared these reference values with previously developed equations for AEX (by gender and race), and found that the ANN models led to the most accurate predictions. When we compared the performance of ANN-based models in derivation/training, internal validation/testing, and external validation random groups, we found that the models based on pooling samples from various geographic areas outperformed the other models (in both central tendency and dispersion of the residuals, ameliorating any cohort effects). In a geographically diverse cohort of subjects with normal spirometry, we computed by both regression and ANN models several predicted equations and z scores for AEX, an alternative measurement of respiratory function. We found that the dynamic nature of the ANN allows for continuous improvement of the predictive models’ performance, thus promising that the AEX could become an essential tool in assessing respiratory impairment.

## Introduction

Interpretation of pulmonary function testing by spirometry relies mainly on comparing measured volumes and flows with their predicted and lower limit of normal (LLN) values. These functional parameters are largely dependent on anthropometric characteristics such as race or ethnicity, gender, age, height and weight. Over the past 50 years, multiple equation sets have been developed and used, generally in separate nominal categories defined by gender and race^1–5^. In current practice, for every lung function measurement (e.g., Forced Vital Capacity, FVC) or calculated variable, values smaller than the 5th percentile (or z scores < − 1.645) of gender and race-referenced healthy individuals define the LLN.

More than four decades ago, an analogue lung function index called the area under the maximum expiratory flow-volume curve (abbreviated AFVx) was computed and proposed for use by Vermaak et al.^6^. In order to describe functional abnormalities, a predicted AFVx based on age, gender and height was computed, and a measured to predicted AFVx ratio was assessed against other established lung function parameters. This ratio appeared to be a sensitive indicator of the degree of lung function impairment^6^. More recently, we published on the utility of a digital functional measurement called Area under Expiratory flow-volume loop (AEX)^7–10^ and its approximations (AEX_1_ through AEX_4_, based on the instantaneous isovolumic flows at 25%, 50% and/or 75% of FVC, or FEF_25_, FEF_50_ and FEF_75_, respectively)^7^ as global tools for diagnosis and severity stratification of respiratory functional impairment. The AEX_1–4_ are good approximations of AEX, and they are especially relevant when the pulmonary function testing software does not provide the actual, measured AEX (as the integral function of flow by variable volume). It is currently unknown if constructs such as predicted AEX_1–4_, which are derived from individual predicted volumes and flows, are useful as surrogates of AEX_predicted_, since FEF_25_, FEF_50_ and FEF_75_ tend to have high inter-test variability (or coefficients of variation), and thus wide confidence intervals for their predicted values. Several authors have also derived and published in the past linear regression-based predictive equations for normal AEX, based on subjects’ age, gender and/or height^6,11^.

In this study, in order to define functional impairments by using AEX, we aimed to find a set of equations for AEX_predicted_ and its z scores (standard deviations) by using artificial neural networks (ANN). The ANN represent a modern computational methodology able to model more complex response surfaces and to circumvent limitations related to fixed equations, variable collinearities, non-gaussian distributions, wide variances and non-linear relationships between predictors. We performed analyses on two groups of normal spirometry tests, one originating from Cleveland, OH (USA), and one from the region of Madrid (Spain), and we compared this approach with optimized regression models using the same variables. The advantage conferred by this approach is that ANN-based models are adaptive and their learning capability could lead to improved predictive performance, thus allowing us to better differentiate between normal and abnormal, and to further define impairments in respiratory physiology.

## Results

**Figure 1:**
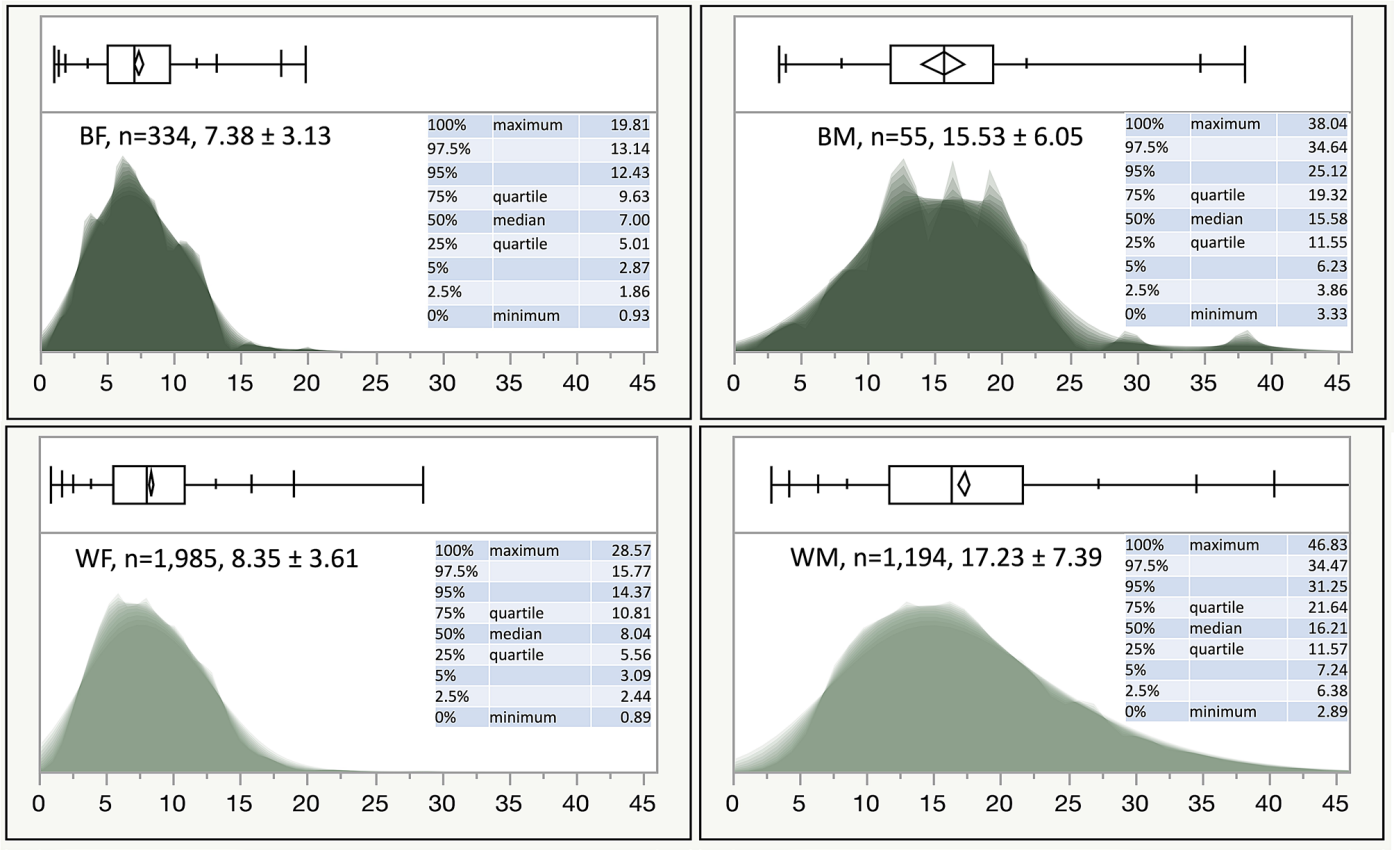
Shadowgrams (overlayed histograms with various X axis bin widths) of AEX by gender and race/ethnicity. To understand the concept of a shadowgram, consider that if the bin width (on the X axis) of a histogram is different, its appearance changes. As such, a shadowgram overlays histograms with different bin widths. Dominant features of the distribution are less transparent on a shadowgram. *B* black, *W* white, *F* females, *M* males. Color codes—dark green: blacks; light green: whites.

**Figure 2:**
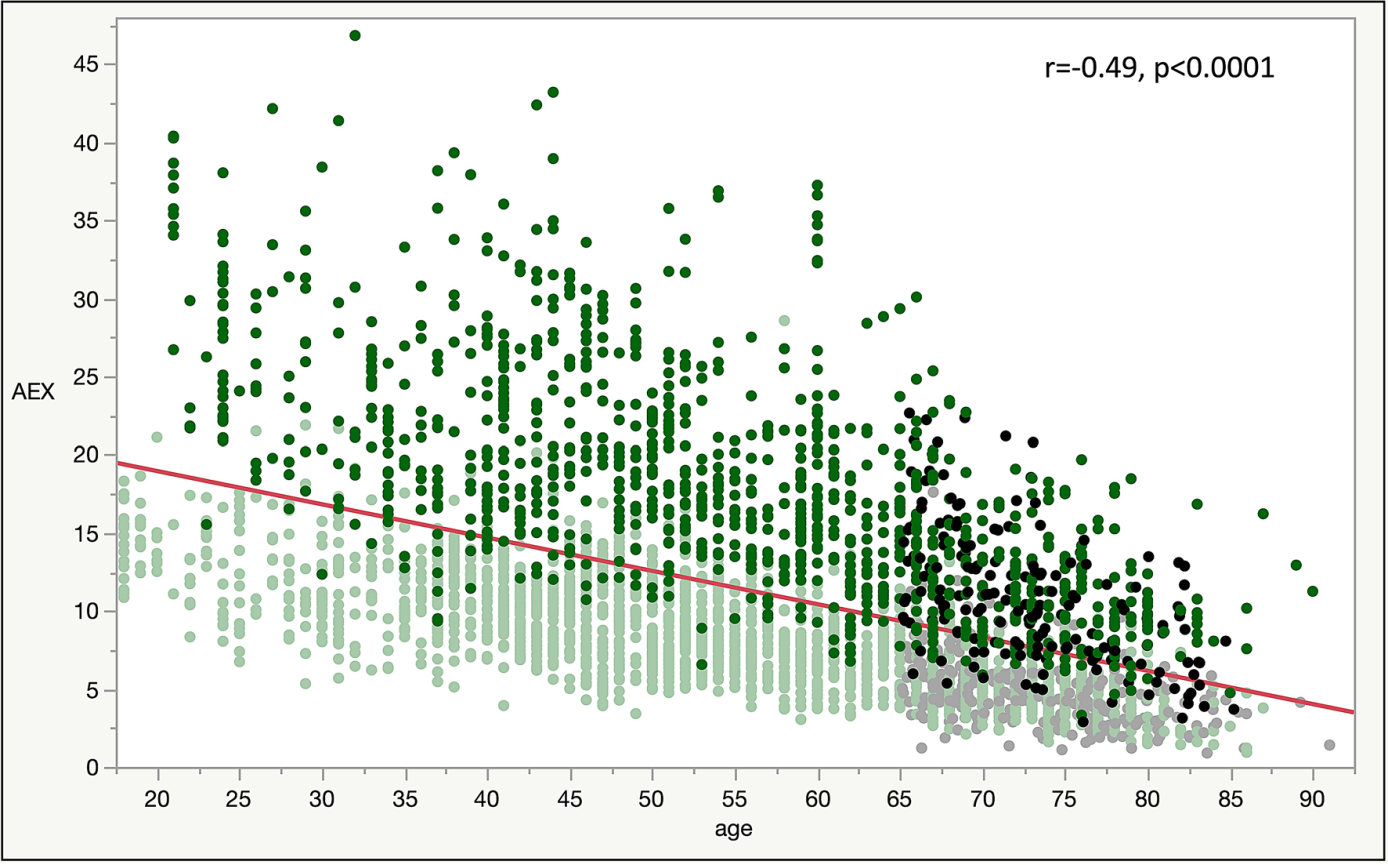
Bivariate linear fit by standard least square method of AEX (L^2^ s^−1^) by age (years). *r* correlation coefficient. Color codes—green: Cleveland group; black/grey: Madrid group. Dark colors (green and black): males; light colors (green and grey): females.

We analyzed 3111 spirometry tests constituting the Cleveland group, which were randomly divided into a derivation/training (66%) and an internal validation/testing set (33%). In this group of tests originating from the USA, approximately 66% of the subjects were women; 87% of the tested individuals were White and 13% self-identified as Black. In addition, we analyzed 457 normal spirometry tests from Spain, which constituted the Madrid group. In this group, 61% were women, and all subjects were characterized as White. The main anthropometric characteristics and pulmonary function measurements of the two groups are shown in Table 1. Figure 1 shows the AEX distributions by gender and race, while Fig. 2 shows the relationship between AEX and the subject’s age at the time of testing.

**Table 1:** Demographic and functional characteristics of the study participants.

	Mean ± standard deviation		Median		25th–75th interquartile range	
	Cleveland Group	Madrid Group	Cleveland Group	Madrid Group	Cleveland Group	Madrid Group
Age (years)	53 ± 15	73 ± 5	53	72	42–65	68–77
Height (cm)	166 ± 10	158 ± 9	165	157	159–173	151–164
Weight (kg)	82 ± 20	70 ± 12	79	70	67–95	62–78
Body Mass Index (BMI, kg/m^2^)	30 ± 7	28 ± 4	29	28	25–33	26–30
PEF (L)	6.8 ± 2.1	5.6 ± 2.0	6.6	5.3	5.3–8.1	4.2–6.8
FEV_PEF_ (L)	0.7 ± 0.3	–	0.6	–	0.5–0.8	–
Estimated FEV_PEF_** (L)	0.7 ± 0.1	0.5 ± 0.1	0.6	0.5	0.5–0.7	0.5–0.6
FEV_1_ (L)	2.8 ± 0.8	2.2 ± 0.6	2.7	2.1	2.2–3.3	1.7–2.6
FEV_1_ z score	− 0.35 ± 0.8	− 0.06 ± 0.9	− 0.46	− 0.15	− 0.95 to 0.15	− 0.70 to 0.63
FEV_1_% pred	95.0 ± 10.8	98.6 ± 15.1	93.8	97.6	87.1–102.0	88.7–109.8
FVC (L)	3.5 ± 1.0	2.8 ± 0.8	3.4	2.7	2.8–4.1	2.2–3.3
FVC z score	− 0.39	− 0.15 ± 0.8	− 0.48	− 0.18	− 0.94 to 0.07	− 0.71 to 0.40
FVC % pred	94.5 ± 10.7	97.4 ± 13.2	93.3	97.0	86.7–101.0	88.0–106.4
FEV_1_/FVC	0.80 ± 0.06	0.78 ± 0.06	0.79	0.78	0.75–0.84	0.74–0.82
FEV_1_/FVC z score	0.09 ± 1.02	0.12 ± 0.99	0.00	0.12	− 0.67 to 0.73	− 0.52 to 0.66
FEV_1_/FVC % pred	100.1 ± 6.7	100.7 ± 7.0	100.0	100.9	95.4–104.7	95.9–105.1
FEF_25_ (L/s)	6.1 ± 1.8	4.8 ± 1.7	5.9	4.6	4.8–7.1	3.6–5.8
FEF_50_ (L/s)	3.4 ± 1.4	2.5 ± 1.1	3.3	2.3	2.5–4.2	1.6–3.2
FEF_75_ (L/s)	1 ± 0.6	0.5 ± 0.3	0.9	0.5	0.6–1.3	0.3–0.7
FEF_25–75_ (L/s)	2.6 ± 1.2	1.7 ± 0.8	2.5	1.5	1.8–3.3	1.1–2.1
AEX (L^2^/s)	11.9 ± 6.8	7.3 ± 4.3	10.4	6.1	7.2–14.8	4.2–9.6
AEX_1_ (L^2^/s)	12.7 ± 7.1	8.3 ± 5.1	11.0	6.9	7.6–15.9	4.8–10.8
AEX_2_ (L^2^/s)	11.4 ± 6.6	–	9.9	–	6.8–14.1	–
AEX_2_* (L^2^/s)	11.6 ± 6.6	7.1 ± 4.3	10.1	5.9	6.9–14.6	4.1–9.5
AEX_3_ (L^2^/s)	10.9 ± 6.3	–	9.4	–	6.4–13.5	–
AEX_3_* (L^2^/s)	11.0 ± 6.3	6.7 ± 4.0	9.5	5.7	6.6–13.9	3.8–8.8
AEX_4_ (L^2^/s)	10.8 ± 6.4	–	9.3	–	6.4–13.4	–
AEX_4_* (L^2^/s)	11.0 ± 6.3	6.6 ± 3.9	9.5	5.6	6.5–13.8	3.7–8.8

*Depicts AEX_2_, AEX_3_ and AEX_4_ obtained based on an Estimated FEV_PEF_**, as this variable was not available in the Madrid group. The formula used was: estimated FEV_PEF_** = 0.157174 + 0.176439*FEV_1_ and was derived in the Cleveland group by modeling the variable based on FEV_1_ only. The coefficient of correlation between actual FEV_PEF_ and Estimated FEV_PEF_** was 0.54, standard deviation of the difference was 0.12, p < 0.0001).

**Figure 3:**
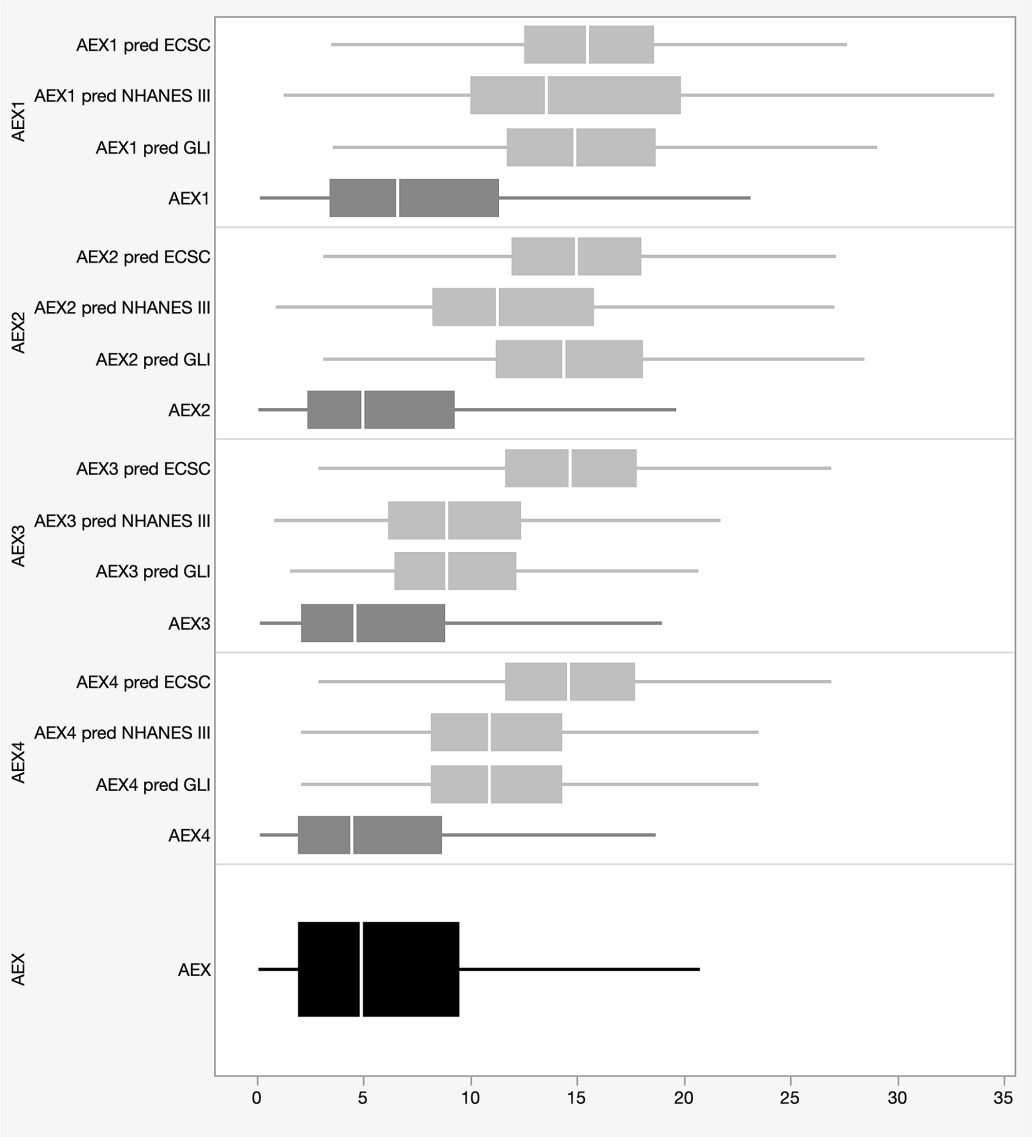
Box-and-whisker plots of AEX and AEX_1_ through AEX_4_ by ECSC, NHANES III and GLI predicted equations (Tukey–Kramer and Welch ANOVA tests: p < 0.0001). *AEX* area under expiratory flow-volume curve, *ECSC* European Community of Steel and Coal, *GLI* global lung initiative, *NHANES III* National Health and Nutrition Examination Survey III.

Next, we computed the AEX approximations called AEX_1_ through AEX_4_ from FVC, Peak Expiratory Flow (PEF), FEF_25_, FEF_50_ and FEF_75_, based on the areas of the triangles and trapezoids delineated by these flows and volumes, as described elsewhere^7^. Then, we compared them with their predicted values, as derived from the main predictive equation sets for FVC, PEF and for the respective isovolumic flows (for the latter, we computed the same triangles and trapezoids’ areas from the predicted values of the instantaneous flows and volumes). For comparison, we used European Community of Steel and Coal (ECSC), National Health and Nutrition Evaluation Survey (NHANES) III and the more recent Global Lung Initiative (GLI) formulas (Fig. 3). The AEX_1_, AEX_2_, AEX_3_ and AEX_4_ approximations of AEX based on one, two, three or four flows, respectively were very close to the actual AEX values (i.e., small deviance and dispersion, Fig. 3—dark grey box plots). First and as iterated before, these approximations are valuable when the pulmonary function software does not provide the actual AEX. All in-between group comparisons showed correlation coefficients > 0.97 and p < 0.0001 (Table 2), findings consistent with our prior investigations^7–10^. Second, we found that predicted AEX_k_ (k = 1–4) based on the major equation sets overestimated on average the actual AEX or its approximations AEX_k_ (k = 1–4)—Fig. 3, light grey box plots. Among the three predicted sets compared, the ECSC equations overestimated the AEX_1_ through AEX_4_ and, indirectly AEX, the most (correlation coefficients were the lowest, i.e., ~ 0.80, p < 0.0001).

**Table 2:** Mean differences (with 95% Confidence Intervals, CI) between actual AEX, AEX approximations (AEX_1_ through AEX_4_) and predicted AEX values by four different formulas (Vermaak et al.^6^; Garcia-Rio et al.^11^, regression and artificial neural networks or ANN, 2020) in the training, testing and validation sets.

Comparison of X vs Y (by actual values, regular linear regression, or standard least squares)	Set	Mean differences (Y–X)	95% CI lower limit	95% CI upper limit	RMSE	RASE	AAE	R^2^	p value
AEX vs AEX_1_	Training	0.613	0.527	0.700	1.865	1.965	1.400	0.911	< 0.0001
	Testing	0.493	0.371	0.616	1.851	1.921	1.387	0.921	< 0.0001
	Validation	0.491	0.363	0.619	1.840	2.016	1.453	0.915	< 0.0001
AEX vs AEX_2_	Training	− 0.493	− 0.531	− 0.455	0.719	0.904	0.665	0.982	< 0.0001
	Testing	− 0.500	− 0.555	− 0.445	0.737	0.924	0.661	0.982	< 0.0001
	Validation	− 0.509	− 0.563	− 0.455	0.749	0.922	0.659	0.983	< 0.0001
AEX vs AEX_3_	Training	− 1.018	− 1.077	− 0.959	1.024	1.561	1.121	0.946	< 0.0001
	Testing	− 1.047	− 1.132	− 0.961	1.074	1.602	1.164	0.946	< 0.0001
	Validation	− 1.066	− 1.157	− 0.976	1.165	1.675	1.168	0.942	< 0.0001
AEX vs AEX_4_	Training	− 1.096	− 1.138	− 1.054	0.574	1.380	1.104	0.958	< 0.0001
	Testing	− 1.097	− 1.155	− 1.040	0.668	1.371	1.106	0.961	< 0.0001
	Validation	− 1.102	− 1.163	− 1.41	0.725	1.406	1.111	0.959	< 0.0001
AEX vs AEX pred Vermaak et al.^6^	Training	4.838	4.665	5.012	2.960	6.117	5.439	0.679	< 0.0001
	Testing	4.669	4.411	4.927	3.014	6.085	5.373	0.674	< 0.0001
	Validation	4.720	4.468	4.973	2.974	6.097	5.393	0.691	< 0.0001
AEX vs AEX pred Garcia-Rio et al.^11^	Training	5.904	5.653	6.154	5.039	8.001	6.675	0.461	< 0.0001
	Testing	5.846	5.482	6.210	5.091	8.030	6.702	0.461	< 0.0001
	Validation	5.821	5.453	6.189	5.220	8.093	6.717	0.458	< 0.0001
AEX vs AEX pred Regression, 2020	Training	0.040	− 0.119	0.198	3.107	3.421	2.521	0.731	< 0.0001
	Testing	− 0.243	− 0.473	− 0.013	3.062	3.483	2.628	0.739	< 0.0001
	Validation	− 0.157	− 0.374	0.059	2.928	3.314	2.420	0.770	< 0.0001
AEX vs AEX pred ANN, 2020	Training	− 0.244	− 0.386	− 0.102	2.734	3.066	2.089	0.785	< 0.0001
	Testing	− 0.470	− 0.674	− 0.267	2.672	3.104	2.175	0.798	< 0.0001
	Validation	− 0.400	− 0.590	− 0.211	2.558	2.925	1.961	0.824	< 0.0001

While the deviance (central tendency) seems slightly larger in the ANN-based model, the dispersion is smaller vs regression-based model using the same parameters (gender, race, age, height and weight), based on RMSE (Root Mean Square Error), RASE (square root of the mean squared prediction error, calculated as the square root of the sum of squares error divided by n) and AAE (average absolute error).

**Figure 4:**
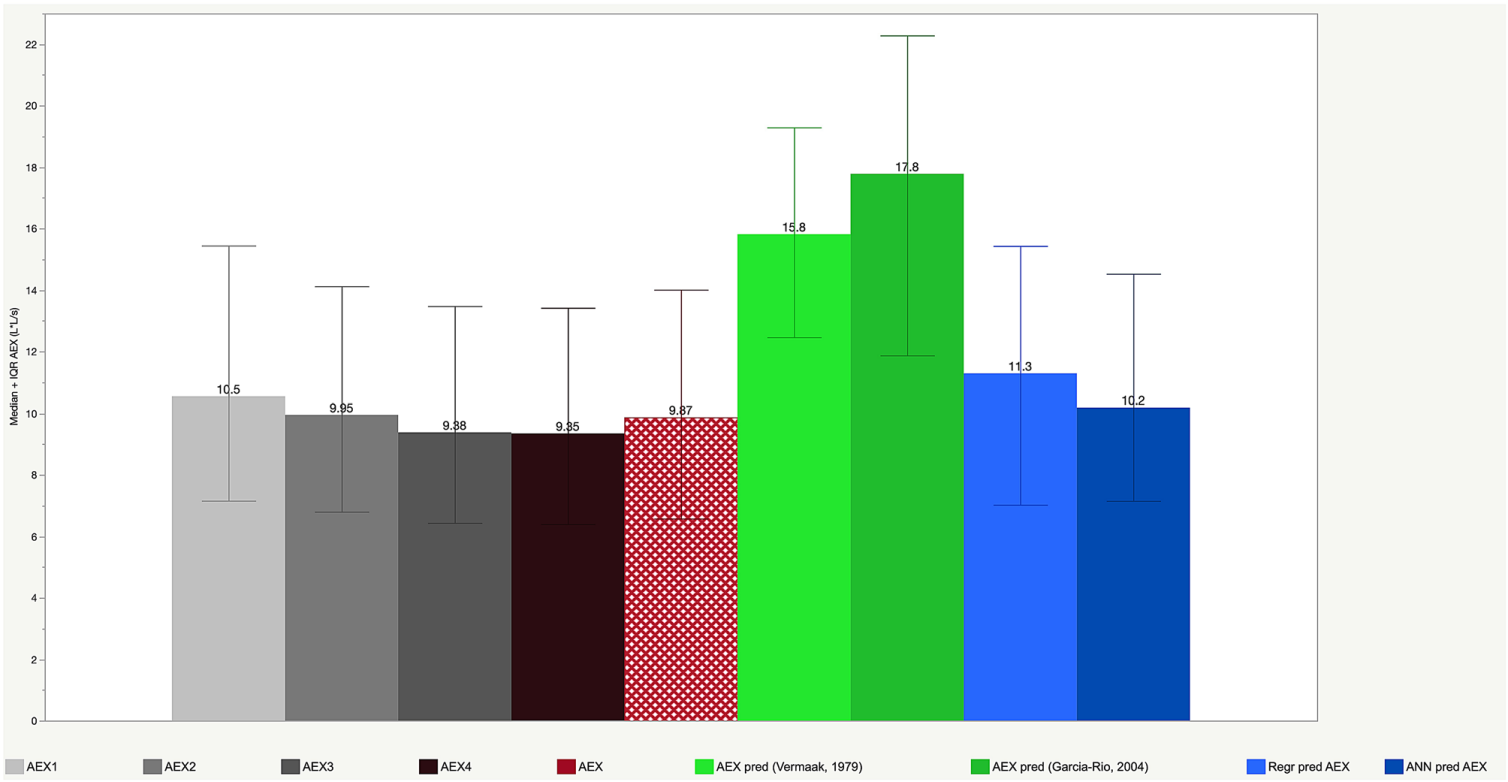
Bar graph showing median values of AEX, AEX_1_ through AEX_4_ and AEX predicted by three different models (Vermaak et al.^6^; Garcia-Rio et al.^11^; and current model, based on artificial neural networks (ANN). Whiskers represent 25th–75th interquartile ranges (IQR).

In a side-by-side bar graph format, Fig. 4 illustrates the median and interquartile ranges (IQR) of AEX_1–4,_ actual AEX and the four predictive models for AEX, i.e., derived from the formulas published by Vermaak et al.^6^, Garcia-Rio et al.^11^, the current linear regression and the ANN-based models. Standard least square-based regression predictive equations for AEX developed de novo in the two groups combined found R^2^ between 0.62 and 0.71, depending on the gender and race-based subset. In these models, weight was a predictive variable only in White men, while race, gender, age, and height remained significant predictors in all the other groups. Regression optimization by transforming the AEX variable for normalization and variance reduction (either by logarithmic or by gamma function transformation), and by using regression regularization techniques (‘generalized regression’) such as ridge penalty regression, single or double lasso (with or without adaptive features), and elastic net led to only minor improvements in Akaike Information Criterion (AICc, maximal delta 2324), generalized R^2^ (maximal delta 0.01, up to 0.75), in Average Absolute Error (AAE, delta 0.24, ~ 2.11) or in the square root of the mean squared prediction error (RASE, likely one of the most important performance measurements here, with maximal delta 0.02, > 3.39) in the random validation subsets of the entire population of tests, by either tenfold crossvalidation or fixed rate holdback validation methods.

**Figure 5:**
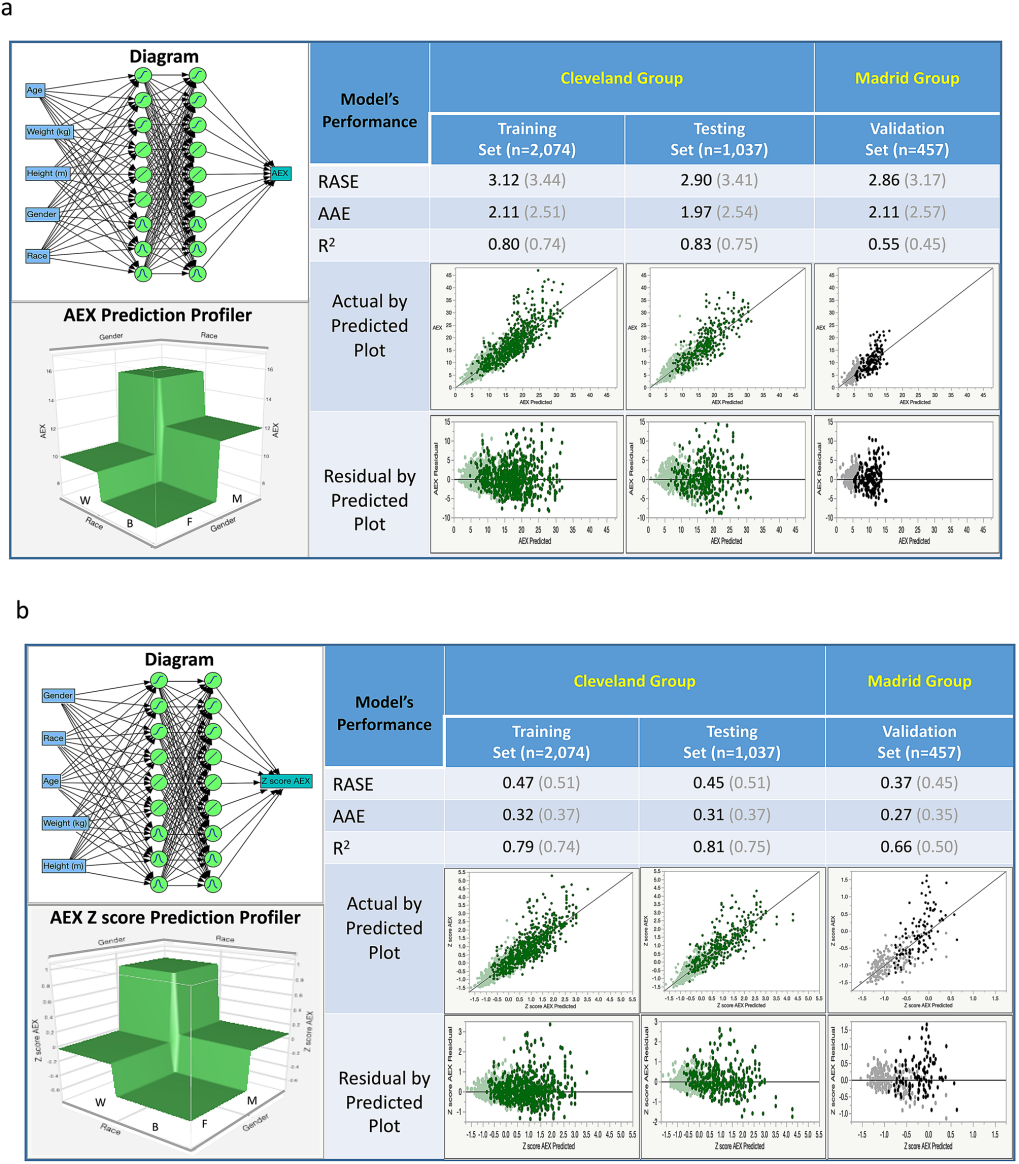
Machine learning (artificial neural network) models predicting AEX (**a**) and AEX z scores (**b**) using as inputs gender, race, age (years), weight (kg) and height (m). Left upper corner shows the model diagram, while left lower corner illustrates the prediction profiler by gender and race. The main table reveals the high performance of the model predicting AEX/Z scores in all subject groups (derivation, internal and external validation sets), with a ‘cohort effect’—a drop in the generalized R^2^ from internal to external validation sets. For comparison, the R^2^, RASE and AAE of the regression model are shown in grey (in brackets). Color codes—green: Cleveland group; black/grey: Madrid group. dark colors (green and black): males; light colors (green and grey): females. *AEX* area under expiratory flow-volume curve, *B* black, *W* white, *F* female, *M* male, *AAE* average of absolute error, *R*^*2*^ R squared statistic, *RASE* square root of the mean squared prediction error, calculated as the square root of the sum of squares error divided by N.

For the ANN, we used as inputs the same parameters, i.e., age, weight, height, gender and race, and the output was AEX or its gender plus race-determined z scores [derived from the formula (X − Mean)/Standard Deviation]. As mentioned earlier (see full details here: Supplemental_Material_S1), the chosen neural network architecture included two ‘hidden’ layers, each containing three sigmodal, three linear and three gaussian activation function nodes. In our analyses, this represented the best architecture in the trade-off between performance and speed, bias and variance, underfitting and overfitting (see also Table 3, which shows the results of ANN ablation experiments). Expectedly, mean predicted AEX was larger in Whites vs. African Americans, and in men vs. women. The ANN–based model predicted the AEX with the highest accuracy, with a median difference of − 0.01 (IQR − 1.66 to 1.30) L^2^/s, and a correlation coefficient of 0.89. The residuals remained low in the external validation lot (Madrid group, Fig. 5a): median difference of − 0.36 (IQR − 1.66 to 1.30) L^2^/s, and a correlation coefficient of 0.76. The model performed well due to its small dispersion, without significant heteroscedasticity, i.e., residuals were not progressively larger at higher values. The model’s R^2^ ranged from 0.80 and 0.83 in the derivation/training and the internal validation/testing sets, and 0.55 in the external validation set (Fig. 5a). These were much higher than prior models’ R^2^ (regression-based), which ranged from 0.39 to 0.42^11^. More importantly, other measurements of model error (Fig. 5a) remained lower vs other regression techniques used. By contrast, in our analyses, the regression-based predicted AEX had a median difference of 0.12 (IQR − 1.90 to 2.03) L^2^/s, and a correlation coefficient of 0.86; in the external validation lot (Madrid group), the median difference was − 1.04 (IQR − 2.73 to 1.21) L^2^/s, and the correlation coefficient was 0.78. Similarly, in our ANN models, the AEX z score prediction, which is important for determining LLN, was also very robust (Fig. 5b). While all inputs were significant independent predictors, the most important factors (total effects, %) for predicted AEX were gender (28.6%), race (28.6%), height (21.6%) and age (20.5%), while for AEX z scores (which are computed by gender and race) were height (50.3%), age (30.7%) and weight (18.8%), respectively.

**Table 3:** Comparison of the Linear Regression (LR) using Standard Least Squares method, Generalized Regression (GR) model using a logarithmic transformation and the double-lasso method, and the main ablation experiments of the Artificial Neural Network (ANN) methods tried.

# Layers	# Hidden nodes	Activation functions			R^2^			Root Mean Square Error (RMSE)			Mean absolute deviance			Average processing time (s)
		Sigmoidal	Gaussian	Linear	Train	Test	Valid	Train	Test	Valid	Train	Test	Valid	
1 (LR)	0				0.731	0.739	0.770	3.107	3.062	2.928	0.040	− 0.243	− 0.157	1
1 (GR)	0				0.757	0.770	0.783	3.261	3.174	3.155	− 0.229	0.035	− 0.993	1
2**	0	2	2	2	0.794	0.780	0.822	3.174	3.221	3.088	2.195	2.308	2.123	100
		2*	2*	2*	0.809	0.787	0.831	3.117	3.192	3.038	2.117	2.273	2.070	240
		4	4	4	0.814	0.784	0.824	3.102	3.269	3.092	2.088	2.289	2.114	120
		4*	4*	4*	0.813	0.789	0.832	3.097	3.196	3.031	2.092	3.196	3.031	660
		6	6	6	0.818	0.789	0.824	3.065	3.178	3.083	2.066	2.262	2.117	150
		6*	6*	6*	0.818	0.797	0.830	3.066	3.144	3.022	2.066	2.222	2.075	1800
3**	6	2	2	2	0.798	0.785	0.822	3.147	3.214	3.091	2.177	2.285	2.126	75
	12	4	4	4	0.810	0.790	0.832	3.088	3.193	3.030	2.110	2.256	2.065	90
	**18**	**6**	**6**	**6**	**0.828**	**0.797**	**0.824**	**3.010**	**3.165**	**3.115**	**2.005**	**2.217**	**2.217**	**180**

The ablation study identified the 2 hidden-layer ANN design (i.e., each layer with three sigmodal, three linear and three Gaussian activation functions) as the best compromise between improved performance and processing speed (bold characters).

*Using an additive sequence of 100 models based on a learning rate of 0.1.

**Using for optimization a robust fit with a squared penalty method and transformed covariates.

**Figure 6:**
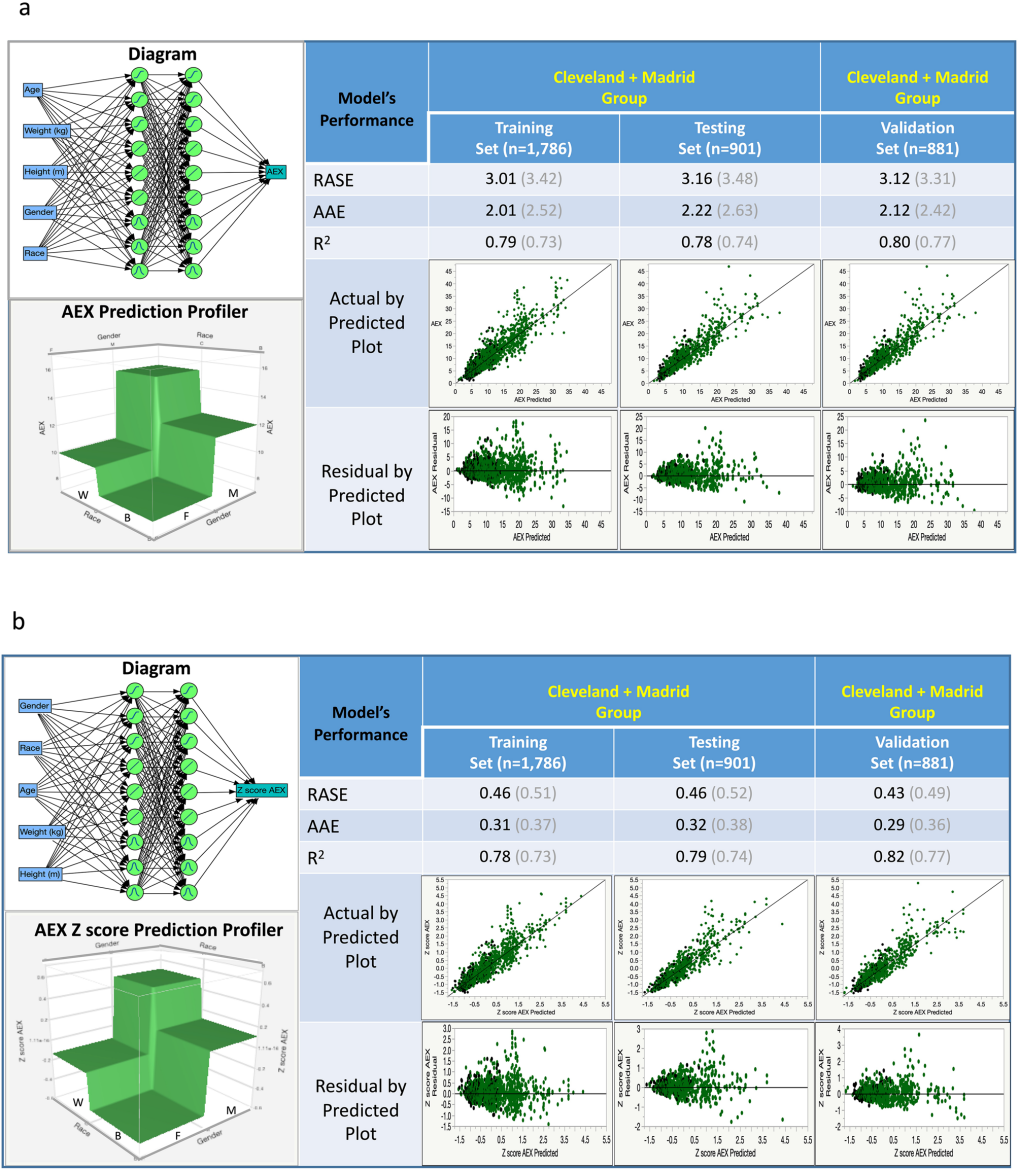
Machine learning (artificial neural network) models predicting AEX (**a**) and AEX z scores (**b**) using as inputs gender, race, age (years), weight (kg) and height (m), with Ongoing Validation. Left upper corner shows the model diagram, while left lower corner illustrates the prediction profiler by gender and race. The main table reveals the high performance of the model predicting both AEX and AEX z scores in all subject groups (derivation and validation sets), without a ‘cohort effect’—a drop in the R^2^ from internal to external validation sets. For comparison, the R^2^, RASE and AAE of the regression model are shown in grey (in brackets). Color codes—green: Cleveland group; black/grey: Madrid group. dark colors (green and black): males; light colors (green and grey): females. *AEX* area under expiratory flow-volume curve, *B* black, *W* white, *F* female, *M* male, *AAE* average of absolute error, *R*^*2*^ R squared statistic, *RASE* square root of the mean squared prediction error, calculated as the square root of the sum of squares error divided by N.

Figures 5 and 6 show two possible modelling approaches by ANN methods. The approach shown in Fig. 5a,b is represented by models developed for AEX_predicted_ and AEX z scores, respectively, on two thirds of the Cleveland group (derivation/training set) and verified on the rest of the subjects (internal validation/testing set), followed by validation (external validation) in the Madrid group. In this case, one can observe the classic ‘cohort effect’, i.e., the model is ‘overfitting’ in the Cleveland group and it loses its precision when applied to *another* cohort, of different subjects. The alternative approach, which is shown in Fig. 6a,b, takes advantage of the adaptability or optimization functions of the ANN models, by mixing the two cohorts and deriving a model on ~ 50% of the subjects, followed by testing in 25% of the cohort (internal validation) and validation on the rest of the tests from the two groups combined. This allowed for better fitting models, in this case with larger R^2^ (0.79–0.82) and improved precision of AEX_predicted_ (consistently lower measurements of error/bias and dispersion). Figures 5 and 6 also show that the condition of homoscedasticity for the models is generally met, i.e., residuals remain roughly in the same range at higher values, with the exception of very few outliers.

In a more comprehensive one-on-one analysis of various variables, Table 2 illustrates the main differences (with 95% Confidence Intervals, CI) between observed AEX, computed AEX_1_ through AEX_4_, predicted AEX values by previously published formulas^6,11^, and by the new regression and ANN-based models.

## Discussion

The main finding of this article is that artificial neural networks (ANN) can provide a great alternative to traditional methodologies in computing normal predicted equations, as well as LLNs based on z scores, in this case applied to Area Under Expiratory flow-volume curve (AEX). The adaptive, machine learning model performed better than a de novo linear regression model (smaller dispersion) and was superior to two previously published equations for AEX^6,11^.

Traditional regression-based models used for deriving predictive equations for pulmonary function have been flawed by internal and external validity biases (‘cohort effects’), or by various degrees of untrue assumptions of normality, additivity or linearity^12^. For these reasons, we used here a more modern method of modelling, able to circumvent collinearities and non-linear relationships, and which can be used in spirometry reference equation derivation, i.e., the ANN. In addition, we found that this methodology outperformed more advanced regression regularization techniques in reducing the bias and the dispersion of the residuals. Nowadays, in an era of exploding computational capabilities, neural networks represent the backbone of many emerging artificial intelligence techniques, which could successfully be applied in our field^13–16^.

We explored first a comparison between measured AEX and its approximations called AEX_1_, AEX_2_, AEX_3_ and AEX_4._ As described before^7^, these parameters are computed based on FVC and PEF (AEX_1_); FVC, PEF and FEF_50_ (AEX_2_); FVC, PEF, FEF_25_ and FEF_75_ (AEX_3_), FVC, PEF, FEF_25_, FEF_50_ and FEF_75_ (AEX_4_). Then we used the most common, validated predictive equations such as ECSC^17,18^, NHANES III^19^ and GLI^2^ sets, stratified by gender and race to derive predicted values for AEX_1_ through AEX_4_.

We illustrate in Fig. 3 several salient findings of our investigation. First, we confirmed our previously published findings^7^, i.e., that AEX_1–4_ are acceptable approximations of AEX (with great metrics of central tendency and dispersion for the estimations). The analyses were performed on a subset of subjects with normal lung function from the Cleveland group (in which inclusion was adjudicated by normal lung volume determinations), and on an external validation set of non-smoking elderly subjects with normal spirometry (the Madrid group). Second, we show that the ECSC equations tend to overestimate these spirometric parameters the most, while GLI-based predicted values for AEX_1_ through AEX_4_ are the closest to the actual normal AEX values.

In Fig. 4 we show both central tendency (medians) and dispersion (IQR) metrics for actual AEX, AEX_1_ through AEX_4_, and for two AEX predicted values, as published before by Vermaak et al.^6^ and Garcia-Rio et al.^11^. Of note, the distribution of these parameters was non-gaussian (sinusoidal or logarithmic-like). In addition to these functional parameters, we included in Fig. 4 the values derived from the linear regression and ANN-based models developed de novo in this article. The ANN-based median AEX_predicted_ (dark blue bar in Fig. 4) was the closest to the actual median AEX (red, double-hashed bar), while the model’s dispersion (as assessed by the IQR) was also the smallest in the ANN-based model. Supplemental Figures S2 and S3 show the distributions of residuals (AEX_predicted_—AEX) by both methods and by gender and race, combining all tests from the two groups. In the figures, highlighted (dark green in Supplemental Figure S2 and dark blue in Supplemental Figure S3) represent the men, while lighter colors illustrate the distributions in women. The linear regression model tended to overestimate AEX in males, while the ANN model provided a more precise estimate of the central tendency in all subgroups. In Table 2, we show the in-between variables’ average differences and their 95% CIs (yet we caution the reader that the residuals are non-normally distributed), together with RMSE (root mean square error), RASE (square root of the mean squared prediction error, calculated as the square root of the sum of squares error divided by n, measurement considered by some as equivalent to an off-sample RSME) and R^2^. As such, we confirmed the high correlations and small dispersions for ANN-based model, both in aggregate and by cohort (for the latter, data not shown).

The ANN-based models described here had as input parameters traditional predictors of lung function, i.e., subjects’ gender, race or ethnicity, height, weight, and age, two layers of nine ‘hidden’ nodes (with three sigmoidal, three linear and three gaussian activation functions), and AEX as the output. The model developed in the Cleveland group was also validated internally—dark green (males) and light green (females) dots, followed by external validation in the Madrid group—black (males) and grey (females) dots, Fig. 5a,b. Expectedly, there was a significant ‘step-down’ in the model’s performance, even when ANN methodology was used and by employing a traditional approach of derivation and internal validation in a population, followed by external validation in *another* cohort. Instead, taking advantage of the learning property of the ANN models (Fig. 6a,b), pooling all tests from the two groups leads to better predictive ability (better central tendency, smaller dispersion and higher percentage of variance explained by the model). See additional online information (link: Supplemental_Material_S1), which also shows the formulas and the code used, for future validation or refinements of the models in other pulmonary function sets.

Several limitations of this investigation deserve to be mentioned. First, the current predictive models for AEX do not consider the intra-individual, test-to-test variability of the AEX measurement, which needs to be explored in future investigations. It is conceivable that, similarly to the large variability of FEF_25_, FEF_50_ and FEF_75_, AEX could also present large variations. This intrinsic variability can be explored and, if found to be high, could potentially be minimized by using AEX variables in concert with other spirometric measurements, approach which can further refine the characterization of the functional impairments. Second, the Madrid cohort included very different subjects, i.e., older, White, and from a small geographic footprint. This limitation could be overcome in the future by extending the geographic coverage and the diversity of the pooled tests. This will allow the ANN models to continue to evolve (trying to the minimize the gradient descent) and to further refine the node equations based on additional variation of the inputs. Third, additional predictors of lung function can be assessed, as modern computational techniques allow us to employ fast and powerful mathematical models, leveraging the unprecedented access to big data, unavailable decades ago, or when using traditional modeling methods. Fourth, one of the disadvantages of the ANN is the complexity of the equations in the hidden nodes, leading to a perceived lack of transparency or ‘black box’ effect, yet it can be visualized easily at each node and in all layers. Fifth, the accuracy of the presented models or equations may not be optimal in a new experimental study that considers different ranges of age, weight and height or other racial profiles. In future training, testing and validation sets, ANN-based models may differ mathematically and deal differently with possible new sources of variance from other factors and with the potential of higher systematic bias. However, this is exactly the point we are making here when we illustrate modeling outcomes in one population with external validation in a different cohort vs ‘pooling’ of all tests together and devising the ANN models that use input variability from all demographic categories. Lastly, the utility of AEX needs to be explored in relationship to specific conditions and outcomes, as most measurements in modern medicine need to be ‘anchored’ against prevention, early diagnosis and development of personalized therapies.

## Conclusion

In this investigation, we used neural network models in a pooled, geographically diverse cohort, in order to compute predicted Area Under Expiratory flow-volume curve, a spirometric measurement that may have great impact on how we define respiratory functional impairment in the future. In a large pool of normal spirometry tests, we found that the learning property of the artificial neural networks allows continuous improvement of the predictive models that compute the reference values for AEX and that these models may outperform traditional methods and validation approaches.

## Methods

Analyses were performed on a development cohort (the Cleveland group) of 3111 consecutive adult subjects who had normal spirometry and normal same-day lung volume testing in the Cleveland Clinic Pulmonary Function Laboratory over a 10-year time span. A second cohort (the Madrid group) was constituted by 457 never-smoker healthy volunteers who met the American Thoracic Society criteria for reference subjects and participated in a Spanish study that was aimed at deriving spirometry reference values for elderly European individuals^11^.

Spirometry was performed and interpreted per the current, joint American Thoracic Society (ATS) and European Respiratory Society (ERS) standards and recommendations^1,20–23^. Lung volume assessments^4^ were performed only in the Cleveland group, by either body plethysmography^24–26^ or helium dilution^27,28^ methods. Normal lung volume testing was defined as values between lower and upper limits of normal for the following parameters: total lung capacity, functional residual capacity and residual volume. All tests were done using a Jaeger-Viasys Master Lab Pro system (Wurzberg, Germany). The most recent, validated and widely applicable reference values, as developed in ‘semi-parametric’ regression-type models and published by the Global Lung Initiative (GLI) were used for spirometry interpretation and definition of normality^2,19^. For lung volumes, the reference values used were those published by Crapo et al.^29^. We did not use the previously published lung volume reference values developed for 65–85 year-old Europeans^30^, as the Cleveland group (the only group with lung volume determinations, which constitute gold standard in pulmonary function testing) was overall younger and likely with different anthropometric characteristics. We calculated the parameters AEX_1_ through AEX_4_ from FVC, FEF_25_, FEF_50_ and FEF_75_, as done elsewhere^7^, and compared them with their predicted values using three of the most popular and widely used equation sets, i.e., European Community for Steel and Coal (ECSC)^18^, National Health and Nutrition Survey (NHANES) III^19^ and Global Lung Initiative (GLI)^2^. The largest AEX was selected from all the pre-bronchodilator spirometry trials performed. In addition, predicted AEX was computed by using two predictive equations for AEX, as published before by Vermaak et al.^6^ and Garcia-Rio et al.^11^.

Statistical analyses were performed using JMP Pro15 (SAS Institute, Cary, NC, USA) and open-access R software (R version 3.6.2, R: A Language and Environment for Statistical Computing, R Core Team, R Foundation for Statistical Computing, Vienna, Austria, 2019, https://www.R-project.org, R Studio 1.2.5033, RStudio, Inc).

Descriptive statistical analysis of available variables was performed. Categorical variables were summarized as frequencies or percentages. Continuous variables were characterized by mean, standard deviation, median and 25^th^–75th interquartile range (IQR), as appropriate (as most distributions were non-gaussian).

The GLI equations^2^ were developed and made available as Generalized Additive Models for Location, Scale and Shape (GAMLSS) in the R software package. The methods are ‘parametric’ in the sense that they require a parametric distribution assumption for the response variables, and ‘semi’ because modelling of the parameters of distribution as functions of exploratory variables may involve non-parametric smoothing functions (link: GAMLSS).

Some of the prior models for pulmonary function normal values used regular linear regression (standard least squares method) by gender and race, relying on predictive variables such as age, height and, occasionally, weight. In this work, regular regression models were improved by several types of optimization approaches, e.g., generalized additive models defining splines for means, variance and skewness (as in the GLI equations^2^), regression regularization techniques such as ridge regression, lasso, elastic net and double lasso techniques, with and without adaptive features, using both native values and logarithmic or gamma transformations (as they represented the closest distribution fits) and comparing them with deep learning algorithms or artificial intelligence (AI) methods. The latter models were based on ANN, which could adjust for more complex relationships and interactions between variables, thus modeling more efficiently complex response surfaces. The machine learning models used here are described in more detail online (link: Supplemental_Material_S1). We tried different ANN architectures, with variable number of nodes (3–5) in the first and second layer, and different activation functions in the hidden nodes. During ablation study experiments, we selected the simplest models that provided the lowest dispersion of the predicted variables (variance) vs smallest bias, and the best trade-off between speed and performance, fitting and overfitting. We used the approach of a derivation (training) and an internal validation (testing) set from the Cleveland group with a random holdback method at 33% rate for the internal validation; following this step, we applied the model on an external validation (validation) set constituted by data points from the Madrid group (Fig. 5a,b). In another approach (Fig. 6a,b), we pooled the data from the two cohorts and developed new ANN-based models; we used a 50–25–25% random partition for training–testing–validation (‘ongoing validation’), respectively. In the AI models used, we performed an analysis of the residuals (i.e., the differences between predicted and actual AEX), checking for normality, internal consistency by various parameters and for homoscedasticity of the residuals. The variables’ weight in various models, independent of the model type and fitting used, was assessed by the dependent resampled inputs methods in JMP Pro15, in which factor values are constructed from observed combinations using a k-nearest neighbors’ approach (k = 5 was used), in order to account for correlation. This method, used mainly when there is an assumption that the inputs (such as height, weight, gender, race and age) are possibly correlated, and treats observed variance and covariance as representative of the covariance structure for the used factors^31^. The performance of the standard least squares fit method (regression) and ANN models were assessed by using the JMP Pro15 platform and comparing the means, the residuals, as well as R^2^, square root of the mean squared prediction error (RASE) and average absolute errors (AAE).

Institutional research oversight approvals were obtained to conduct the study and to waive subjects’ informed consent (Cleveland Clinic IRB EX#0504/EX#19-1129; Emory IRB# 00049576/Atlanta VA R&D Ioachimescu-002; and Ethics Committee of the University Hospital of La Paz HULP #PI-70).

### Ethics

These analyses were performed were performed in accordance with the relevant rules, guidelines and regulations (and regulatory approvals obtained from Institutional Review Boards).

### Informed consent

No informed consent was necessary, as these were data analyses of existing databases.

## Supplementary information

Supplementary Legends.

Supplementary Information.

Supplementary Figure S2.

Supplementary Figure S3.

## Abbreviations

AAE: Average absolute error
AEX: Area under expiratory flow-volume curve
AEX_k_: Area under expiratory flow-volume curve approximation based on number = k isovolumic flows
AFVx: Area under maximum expiratory flow-volume curve
AI: Artificial intelligence
ANN: Artificial neural network
ATS: American Thoracic Society
BMI: Body mass index
CI: Confidence intervals
ECSC: European Community for Steel and Coal
ERS: European Respiratory Society
FDR: False discovery rate
FEF_25_, FEF_50_ and FEF_75_: Forced expiratory flow at 25, 50 and 75% of forced vital capacity
FEV: Forced expiratory volume
FEV_1_: Forced expiratory volume in 1 s
FVC: Forced vital capacity
GLI: Global lung initiative
HSD: (Tukey–Kramer) honest significant differences test
IQR: Inter-quartile range
IRB: Institution Review Board
NHANES III: National Health and Nutrition Survey 3
PEF: Peak expiratory flow
RASE: Square root of the mean squared prediction error (square root of [sum of square error divided by number of observations])
