# Supplementary material for: Area under the expiratory flow-volume curve: predicted values by artificial neural networks
Source: Sci Rep. 2020 Oct 6;10:16624. doi: 10.1038/s41598-020-73925-0 (PMC7538954; doi:10.1038/s41598-020-73925-0)
Supplement: Supplementary file 1 — Supplementary Legends. [file 41598_2020_73925_MOESM1_ESM.docx]

**Supplemental Material S1 (online).**

Neural network methodology and an example of the codes used for the two Artificial Neural Network (ANN) models, as shown in Figures 6A and 6B.

**Online Figure S2.**

A Kernel Density Estimation (KDE) diagram is a graphic representation of a non-parametric density smoothing fit model. The figure illustrates KDE smooth curves superimposed on shadowgrams of the residuals by Regression (A) and by Neural Networks (B). The bandwidth (or smoothing parameter h) was optimized to minimize the mean integrated squared error.

Color codes - dark green: Males, light green: Females.

**Online Figure S3.**

The figure shows KDE smooth curves superimposed on shadowgrams of the residuals by Regression (top) and Neural Networks (bottom), by race or ethnicity.

Color codes - dark blue: Males, light blue: Females.

Abbreviations: AEX: Area Under Expiratory flow-volume curve; B: Black; W: White
