# Supplementary material for: Area under the expiratory flow-volume curve: predicted values by artificial neural networks
Source: Sci Rep. 2020 Oct 6;10:16624. doi: 10.1038/s41598-020-73925-0 (PMC7538954; doi:10.1038/s41598-020-73925-0)
Supplement: Supplementary file 2 — Supplementary Information. [file 41598_2020_73925_MOESM2_ESM.docx]

Title:

**Area Under the Expiratory Flow-Volume Curve: Predicted Values by Artificial Neural Networks**

Authors:

**Octavian C. Ioachimescu, MD, PhD^1^**

**James K. Stoller, MD, MS^2^**

**Francisco Garcia-Rio, MD, PhD^3^**

**Affiliations:**

^1^Professor of Medicine, Division of Pulmonary, Allergy, Critical Care and Sleep Medicine, School of Medicine, Emory University, Atlanta VA Sleep Medicine Center, 250 N Arcadia Ave, Decatur, GA 30030; telephone: (404) 321-6111-207258, fax: (404) 417-2903, e-mail: [oioac@yahoo.com](mailto:oioac@yahoo.com)

^2^Jean Wall Bennett Professor of Medicine, Chair - Education Institute, Cleveland Clinic, 9500 Euclid Ave Cleveland, Ohio, telephone: (216) 444-1960, fax: (216) 445-8160, e-mail: [Stollej@ccf.org](mailto:Stollej@ccf.org)

^3^Professor of Pulmonology, Servicio de Neumología, Hospital Universitario La Paz, IdiPAZ – Departamento de Medicina, Universidad Autónoma de Madrid – Centro de Investigación Biomédica en Red en Enfermedades Respiratorias (CIBERES), Madrid, Spain, e-mail: [francisco.garcia@uam.es](mailto:francisco.garcia@uam.es)

**Correspondence to:**

Octavian Ioachimescu, MD, Ph.D., Atlanta VA Sleep Medicine Center, 250 N Arcadia Ave, Decatur, GA 30030; telephone: (404) 321-6111-207258, fax: (404) 417-2903, e-mail: [oioac@yahoo.com](mailto:oioac@yahoo.com)

**Supplemental Material S1**

**Neural Network Methodology (see also:** [**JMP Pro 15 Manual**](https://www.jmp.com/content/dam/jmp/documents/en/support/jmp15/predictive-and-specialized-modeling.pdf)**)**

The Artificial Neural Network (ANN) platform in JMP Pro15 implements a fully connected multi-layer perceptron with one or two layers. *In the analyses performed for predicted AEX and AEX Z scores, we used 2 (two) hidden layers.*

The functions applied at the nodes of the hidden layers are called activation functions, which are transformations of a linear combination of the X variables (inputs). *We used the following activation functions, with 3 (three) nodes each:*

1. TanH (hyperbolic tangent), which is a sigmoid function, transforms values to be between -1 and 1, and is the centered and scaled version of the logistic function: (e^2x^ – 1)/(e^2x^ + 1);
2. Linear (identity function), in which the linear combination of the X variables is not transformed;
3. Gaussian (radial), for radial basis function behavior or when the response surface is Gaussian (normal) in shape: $e^{-x^{2}}$,

where x is a linear combination of the X variables.

**Boosting:** Boosting is the process of building a large additive neural network model by fitting a sequence of smaller models. Each of the smaller models is fit on the scaled residuals of the previous model. The models are combined to form the larger final model. The process uses validation to assess how many component models to fit, not exceeding the specified number of models. Boosting is often faster than fitting a single large model. However, the base model should be a 1 to 2 node, single-layer model. The benefit of faster fitting can be lost if a large number of models is specified. *No boosting was used in the current models.*

**Fitting options:**

**Transform Covariates** - Transforms all continuous variables to near-normality using either the Johnson Su or Johnson Sb distribution. Transforming the continuous variables helps mitigate the negative impact of the outliers or heavily skewed distributions. *We did use this option*.

**Robust Fit** - Trains the model using least absolute deviations instead of least squares. This option is useful if one wants to minimize the impact of response outliers. This option is only available for continuous responses. *We did use this feature*.

**Penalty Method** - To mitigate the tendency of the ANN to overfit data, the fitting process incorporates a penalty on the likelihood. The penalty is λp(β_i_), where λ is the penalty parameter, and p( ) is a function of the parameter estimates, called the penalty function. Validation is used to find the optimal value of the penalty parameter. *We used the squared penalty function*:

∑β_i_^2^ ,

which is a method appropriate when the assumption that most of the X variables contribute to the predictive ability of the model.

**Number of Tours** - Specify the number of times to restart the fitting process, with each iteration using different random starting points for the parameter estimates. The iteration with the best validation statistic is chosen as the final model. *We assessed the performance of the models with 20-100 tours.*

**Validation Method:**

Neural networks are very flexible models, hence they have a tendency to overfit data, i.e., the model predicts the fitted data very well, but predicts future observations poorly. To mitigate overfitting, the JMP Pro15 ANN platform offers =several options:

- applies a penalty on the model parameters;
- uses an independent data set to assess the predictive power of the model (cross-validation).

Validation is the process of using part of a data set to estimate model parameters, and using the other part to assess the predictive ability of the model:

- The training set is the part that estimates model parameters.
- The validation set is the part that estimates the optimal value of the penalty, and assesses or validates the predictive ability of the model.
- The test set is a final, independent assessment of the model’s predictive ability *(we used an external validation set for further verification of the model performance)*.

The training, validation, and test sets are created by sub-setting the original data into parts by various methods (Holdback, K-fold or Excluded Row Holdback).

The Holdback method randomly divides the original data into training and validation sets.

*We used a Holdback method at a rate of 0.33 (2:1=training:validation).*

**Random Seed** - specifies a nonzero numeric random seed in order to reproduce the same validation assignment for future launches of the ANN platform. *We used a random seed of 1234.*

**Informative Missing** – this feature enables informative coding of missing values, i.e., estimation of a predictive model despite data missingness. For a continuous variable, missing values are replaced by the mean of the variable and a missing value indicator, named ‘Is Missing’, is created and included in the model. If a variable is transformed using the Transform Covariates fitting option on the Model Launch control panel, missing values are replaced by the mean of the transformed

variable. For a categorical variable, missing values are treated as a separate level of that variable.

*Despite minimal data missingness, we used this feature.*

**Model Report**

A model report is created for every neural network model. Measures of fit are generated for both the training and the validation sets.

**Generalized R^2^** - a measure that can be applied to general regression models, based on the likelihood function L and scaled to have a maximum value of 1 (perfect model) to a minimum of 0 (a model no better than a constant model). The Generalized R^2^ measure simplifies to the traditional R^2^ for continuous normal responses in the standard least squares setting. Generalized R^2^ is also known as the Nagelkerke or Craig and Uhler R^2^ , which is a normalized version of Cox and Snell’s pseudo R^2^ (Nagelkerke, N. J. D, “A Note on a General Definition of the Coefficient of Determination”, Biometrika 1991, 78:691–692).

**Entropy R^2^** (appears only when the response is nominal or ordinal) - measure of fitthat compares the log-likelihoods from the fitted model and the constant probability

model. Entropy RSquare ranges from 0 to 1, where values closer to 1 indicate a better fit.

**Root Mean Square Error** (RMSE, ~Standard Deviation) - When the response is nominal or ordinal, the differences are between 1 and p (the fitted probability for the response level that actually occurred).

**Mean Abs Dev** - the average of the absolute values of the differences between the response and the predicted response. When the response is nominal or ordinal, the differences are between 1 and p (the fitted probability for the response level that actually occurred).

**Misclassification Rate** - the rate for which the response category with the highest fitted probability is not the observed category (used only when the response is nominal or ordinal).

**-LogLikelihood** - the negative of the log-likelihood (same as in fitting linear models).

**SSE** - the error sums of square (available only when the response is continuous).

**Sum Freq** - the number of observations used.

**Example of an AEX ANN model (shown in Figure 6A):**

/* Neural SAS Scoring*/

/*%PRODUCER: JMP - Neural */

/*%TARGET: AEX */

/*%INPUT: Gender */

/*%INPUT: Race */

/*%INPUT: Age */

/*%INPUT: Height_m_ */

/*%INPUT: Weight_kg_ */

/*%OUTPUT: AEX_Predicted */

LABEL AEX_Predicted = 'Predicted: AEX';

/* Transformation Code */

AgeTran = log((Age-13.1397179282554)/(92.0438356164384-Age ));

Height_m_Tran = log((Height_m_-1.31)/(2.02-Height_m_ ));

Weight_kg_Tran = log((Weight_kg_-33.7543067904936)/(192.797570497454-Weight_kg_ ));

/* Hidden Layer Code */

H1 = tanh(.5*(-2.46456333020668*((Gender="F")-(Gender="M")) + 1.47967591307273*((Race="B")-(Race="C")) + 1.65991522852929*AgeTran + 4.06155071934236*Height_m_Tran + 0.635764733599216*Weight_kg_Tran + 3.59323269429653));

H2 = tanh(.5*(2.61396557945957*((Gender="F")-(Gender="M")) + 0.771383044867249*((Race="B")-(Race="C")) + 1.21060197630704*AgeTran + -0.334239581989715*Height_m_Tran + 1.90353379816768*Weight_kg_Tran + 4.84376539962645));

H3 = tanh(.5*(-1.45603900164785*((Gender="F")-(Gender="M")) + 2.59449676741143*((Race="B")-(Race="C")) + -3.76633533350477*AgeTran + 3.61171728766646*Height_m_Tran + 2.07783789470776*Weight_kg_Tran + 4.4129241034839));

H4 = 1.67882717243781*((Gender="F")-(Gender="M")) + 0.681165547422294*((Race="B")-(Race="C")) + -0.0771357185523173*AgeTran + -2.76630323770595*Height_m_Tran + -0.0938751836353611*Weight_kg_Tran + -1.09312800781588;

H5 = -1.00969820323109*((Gender="F")-(Gender="M")) + -3.55097532062384*((Race="B")-(Race="C")) + 3.24977347191048*AgeTran + -0.997378018051525*Height_m_Tran + -1.80406405308477*Weight_kg_Tran + -3.43428147979851;

H6 = -0.965888709771627*((Gender="F")-(Gender="M")) + 4.54415137549724*((Race="B")-(Race="C")) + 1.76983752099036*AgeTran + 3.47689295311732*Height_m_Tran + 1.61825760457943*Weight_kg_Tran + 4.12434937206621;

H7 = exp(-0.5*(-1.76321820349081*((Gender="F")-(Gender="M")) + -4.9096589820819*((Race="B")-(Race="C")) + -0.482503028084517*AgeTran + 0.448677318494373*Height_m_Tran + 0.0725878098229275*Weight_kg_Tran + -0.165883950046146)**2);

H8 = exp(-0.5*(1.4788365770759*((Gender="F")-(Gender="M")) + 2.00130070179727*((Race="B")-(Race="C")) + -2.93662742606168*AgeTran + -0.171767805840719*Height_m_Tran + -1.90955637712428*Weight_kg_Tran + -3.89844604954237)**2);

H9 = exp(-0.5*(-1.3020663202178*((Gender="F")-(Gender="M")) + -0.116933615795906*((Race="B")-(Race="C")) + -1.78426622070762*AgeTran + -1.03030321689587*Height_m_Tran + -0.836131292284031*Weight_kg_Tran + -0.29553602455338)**2);

HH1 = tanh(.5*(5.09473029096139*H1 + 0.788132352489788*H2 + -1.9794747980769*H3 + 0.638270700061095*H4 + -0.0353855983742029*H5 + 0.186315310743916*H6 + -5.17558158646679*H7 + -1.543255124954*H8 + -4.03223861831512*H9 + 1.12549858608522;));

HH2 = tanh(.5*(3.94796124999324*H1 + -0.666748164702386*H2 + 2.49118854402224*H3 + -1.78682714652306*H4 + 0.178634918515022*H5 + 0.759783737807044*H6 + 0.422467686168871*H7 + 3.6309562337404*H8 + 1.16113737445423*H9 + 1.69898151981389;));

HH3 = tanh(.5*(8.30657941960245*H1 + -0.841001575226737*H2 + 6.16972422847742*H3 + 0.151859631007362*H4 + -2.4453580355827*H5 + -2.4444050340478*H6 + 1.26776598844394*H7 + -1.05204740518326*H8 + -6.84506423278312*H9 + 0.44350625565605;));

HH4 = 2.1438526898475*H1 + -1.42558486618026*H2 + -0.699661508918816*H3 + -0.667746737367524*H4 + -0.486970601472436*H5 + 0.414859528161936*H6 + 5.78236654639777*H7 + 1.11273407600801*H8 + 1.28848411221832*H9 + 2.13235691408264;;

HH5 = -1.76974552837415*H1 + 0.174091912794293*H2 + -0.0140344659432909*H3 + 1.24909289938003*H4 + -2.30865024714953*H5 + 1.55813230759294*H6 + -0.921800149160203*H7 + -0.210470410885751*H8 + 0.11499152664727*H9 + -0.227802757056246;;

HH6 = -0.840267768596526*H1 + 0.851426898175675*H2 + 1.52270205616236*H3 + 0.576832287547683*H4 + 0.0262788464019145*H5 + 1.18880214840805*H6 + -0.923785794754798*H7 + -0.513689971049726*H8 + 1.92424325589105*H9 + 1.59472341407688;;

HH7 = exp(-0.5*(2.2173538682083*H1 + -3.79577356298194*H2 + -9.42879454383249*H3 + -1.50894821210487*H4 + 0.691729109918117*H5 + -3.00364521016515*H6 + 1.27349607849308*H7 + 1.07212447514632*H8 + 14.7411133137567*H9 + -2.88137425123901;)**2);

HH8 = exp(-0.5*(0.889346718335607*H1 + -0.794212544709894*H2 + -2.61465569878563*H3 + 2.44903402288199*H4 + 0.758030258093762*H5 + 0.578949518796636*H6 + -2.24001129666061*H7 + 4.77063298422831*H8 + -4.07080801649444*H9 + 0.993012689547749;)**2);

HH9 = exp(-0.5*(-0.31668251631798*H1 + 0.667938388430089*H2 + 0.0797785769220005*H3 + 0.891036728834772*H4 + 3.99648671158583*H5 + -2.71714290520455*H6 + 0.892885824199243*H7 + -0.246251605021248*H8 + 2.8043741277139*H9 + 0.333755933931781;)**2);

/* Final Layer Code */

THETA1=-2.53250502038566*HH1 + -2.03687931789572*HH2 + 1.31877131864866*HH3 + 1.57262362054443*HH4 + -0.0765911846440715*HH5 + -0.631957715935782*HH6 + 5.51643621989536*HH7 + -2.10101637658033*HH8 + -3.85039900638056*HH9 + 8.23022812055414;

/* Response Mapping Code */

AEX_Predicted = THETA1;

**Example of an AEX z score ANN model (shown in Figure 6B):**

/* Neural SAS Scoring*/

/*%PRODUCER: JMP - Neural */

/*%TARGET: Z_score_AEX */

/*%INPUT: Gender */

/*%INPUT: Race */

/*%INPUT: Age */

/*%INPUT: Height_m_ */

/*%INPUT: Weight_kg_ */

/*%OUTPUT: Z_score_AEX_Predicted */

LABEL Z_score_AEX_Predicted = 'Predicted: Z_score_AEX';

/* Transformation Code */

AgeTran = log((Age-13.1397179282554)/(92.0438356164384-Age ));

Height_m_Tran = log((Height_m_-1.31)/(2.02-Height_m_ ));

Weight_kg_Tran = log((Weight_kg_-33.7543067904936)/(192.797570497454-Weight_kg_ ));

/* Hidden Layer Code */

H1 = tanh(.5*(1.64084014792183*((Gender="F")-(Gender="M")) + -1.12845147220577*((Race="B")-(Race="C")) + 0.161865447119676*AgeTran + -1.73906860867568*Height_m_Tran + -0.999529049616323*Weight_kg_Tran + -0.0363863034798198));

H2 = tanh(.5*(1.5656965049099*((Gender="F")-(Gender="M")) + 2.07185282621095*((Race="B")-(Race="C")) + 2.3104564908942*AgeTran + -0.561760553910609*Height_m_Tran + 1.08316024334008*Weight_kg_Tran + 2.71012404887367));

H3 = tanh(.5*(0.158772643908697*((Gender="F")-(Gender="M")) + -2.65428543040728*((Race="B")-(Race="C")) + -1.19179814842256*AgeTran + 0.744548727328135*Height_m_Tran + 3.36427609797232*Weight_kg_Tran + 1.03979058171971));

H4 = -1.17325096732341*((Gender="F")-(Gender="M")) + -2.93632633095433*((Race="B")-(Race="C")) + 1.2572245310755*AgeTran + 0.318096040298904*Height_m_Tran + 0.693006786434321*Weight_kg_Tran + 0.360780177654219;

H5 = -0.63216180534446*((Gender="F")-(Gender="M")) + -2.85822993054269*((Race="B")-(Race="C")) + -0.245747219490349*AgeTran + -2.66107153380856*Height_m_Tran + 1.38680219699886*Weight_kg_Tran + -0.158061538741803;

H6 = 0.671505877112182*((Gender="F")-(Gender="M")) + 2.62553744619736*((Race="B")-(Race="C")) + -0.388981222484197*AgeTran + -0.721147569434196*Height_m_Tran + -0.715931316518569*Weight_kg_Tran + 1.14946775105686;

H7 = exp(-0.5*(1.77251881448732*((Gender="F")-(Gender="M")) + 0.713443130478113*((Race="B")-(Race="C")) + -1.29065669051656*AgeTran + -0.443713616582367*Height_m_Tran + -0.852476126409007*Weight_kg_Tran + 2.35437233748141)**2);

H8 = exp(-0.5*(0.519638087126661*((Gender="F")-(Gender="M")) + 3.74458488195776*((Race="B")-(Race="C")) + 1.48147851925102*AgeTran + -0.256548505837074*Height_m_Tran + -2.47434930058245*Weight_kg_Tran + -1.92863539128138)**2);

H9 = exp(-0.5*(-1.23923694037766*((Gender="F")-(Gender="M")) + 3.30646479869235*((Race="B")-(Race="C")) + 2.58151171551031*AgeTran + -0.508124132016068*Height_m_Tran + 0.987272577408515*Weight_kg_Tran + 6.52956677054785)**2);

HH1 = tanh(.5*(-0.35212720295182*H1 + -0.382185526244358*H2 + 1.42894991010768*H3 + -0.785777275559363*H4 + -0.328082224248225*H5 + 0.150823342515495*H6 + 0.0589864490125253*H7 + 1.77698175764553*H8 + -0.591621038503721*H9 + -0.0449836163048655;));

HH2 = tanh(.5*(1.95368541390929*H1 + -1.89183698671398*H2 + 0.148558463331868*H3 + -0.55073624189798*H4 + 0.477842059136044*H5 + -0.543526259609111*H6 + 1.69313038282816*H7 + -0.330553813073025*H8 + -0.30786686953773*H9 + 1.09930724440203;));

HH3 = tanh(.5*(-1.25047862288395*H1 + 0.750599620862755*H2 + -1.28877201984461*H3 + 1.76099492719964*H4 + 1.2161421925479*H5 + -0.802386678289541*H6 + 0.365612682746399*H7 + -0.0309628276836114*H8 + 1.2725757202638*H9 + 0.0386366928380105;));

HH4 = -1.51630786840959*H1 + -1.44744724913026*H2 + -0.966671855991383*H3 + 0.385595536047723*H4 + -0.535818051426725*H5 + -0.129725825516199*H6 + -1.17389490916628*H7 + 0.243591015997878*H8 + 0.163541498321764*H9 + 0.372334873397476;;

HH5 = -0.286573473835107*H1 + -0.874378281892591*H2 + 0.0182784188092604*H3 + -0.361669382974201*H4 + 1.41089028146289*H5 + 0.799776453920579*H6 + -0.317455552508142*H7 + 0.759341598869141*H8 + 0.0540253434627338*H9 + 1.1030241257998;;

HH6 = -1.92216042361484*H1 + 0.368910546264871*H2 + -0.237147082283965*H3 + -0.282186060857773*H4 + -0.204597989400608*H5 + -0.674225318089979*H6 + -0.476431977821807*H7 + -0.627789018596059*H8 + -0.859098233957378*H9 + 0.421317136520343;;

HH7 = exp(-0.5*(-0.86310270798291*H1 + -0.719571261658165*H2 + -2.7402746178043*H3 + -1.611965477779*H4 + -0.947149075513304*H5 + 0.418993584436025*H6 + 3.23205799816656*H7 + 1.35382156633952*H8 + 1.89795852374957*H9 + 0.369735901251502;)**2);

HH8 = exp(-0.5*(-0.113096475913099*H1 + 0.0788370535880113*H2 + -0.406473465733938*H3 + 1.45245326457133*H4 + -0.365993233690041*H5 + 1.11564599924492*H6 + 1.51534999211069*H7 + 2.19715103000515*H8 + -0.935265298146625*H9 + 0.0703472057129823;)**2);

HH9 = exp(-0.5*(1.98324238318105*H1 + 2.40918370224432*H2 + 0.851500161286735*H3 + 1.51674943197955*H4 + 1.36754738175766*H5 + 0.551025475457369*H6 + -0.218746494880826*H7 + -0.440544198531233*H8 + 0.815724948573559*H9 + -0.55719039970036;)**2);

/* Final Layer Code */

THETA1=1.45092217904273*HH1 + 0.723653975956498*HH2 + 1.03133010149866*HH3 + 0.205451635651191*HH4 + 0.0264023918266309*HH5 + 0.280996326602454*HH6 + 0.0498701098029205*HH7 + 0.297989564362116*HH8 + 0.512914643407602*HH9 + 0.214692146308569;

/* Response Mapping Code */

Z_score_AEX_Predicted = THETA1;
