# Supplementary figures and images for: Area under the expiratory flow-volume curve: predicted values by artificial neural networks
Source: Sci Rep. 2020 Oct 6;10:16624. doi: 10.1038/s41598-020-73925-0 (PMC7538954; doi:10.1038/s41598-020-73925-0)

## Slide 1
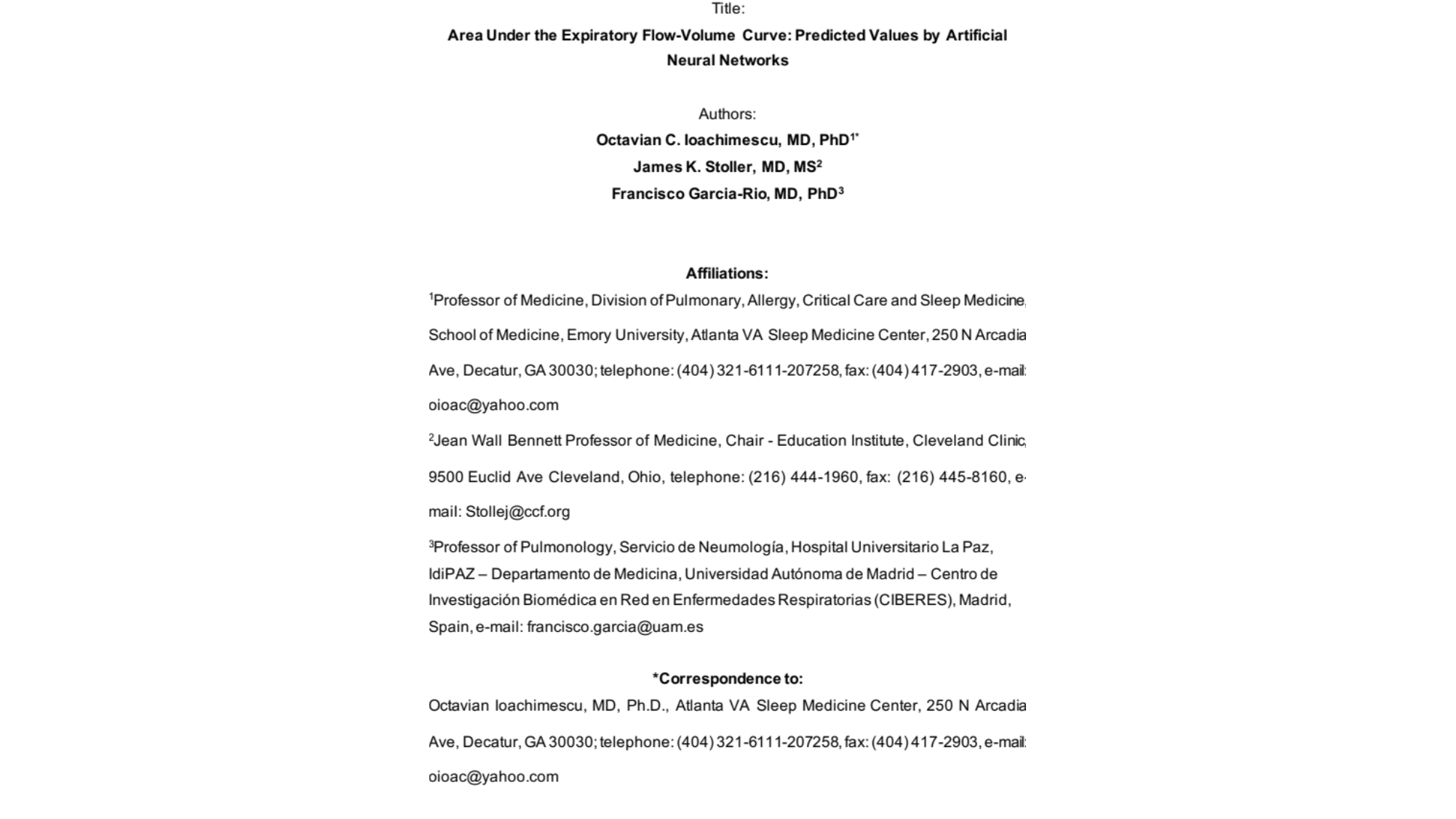

## Slide 2
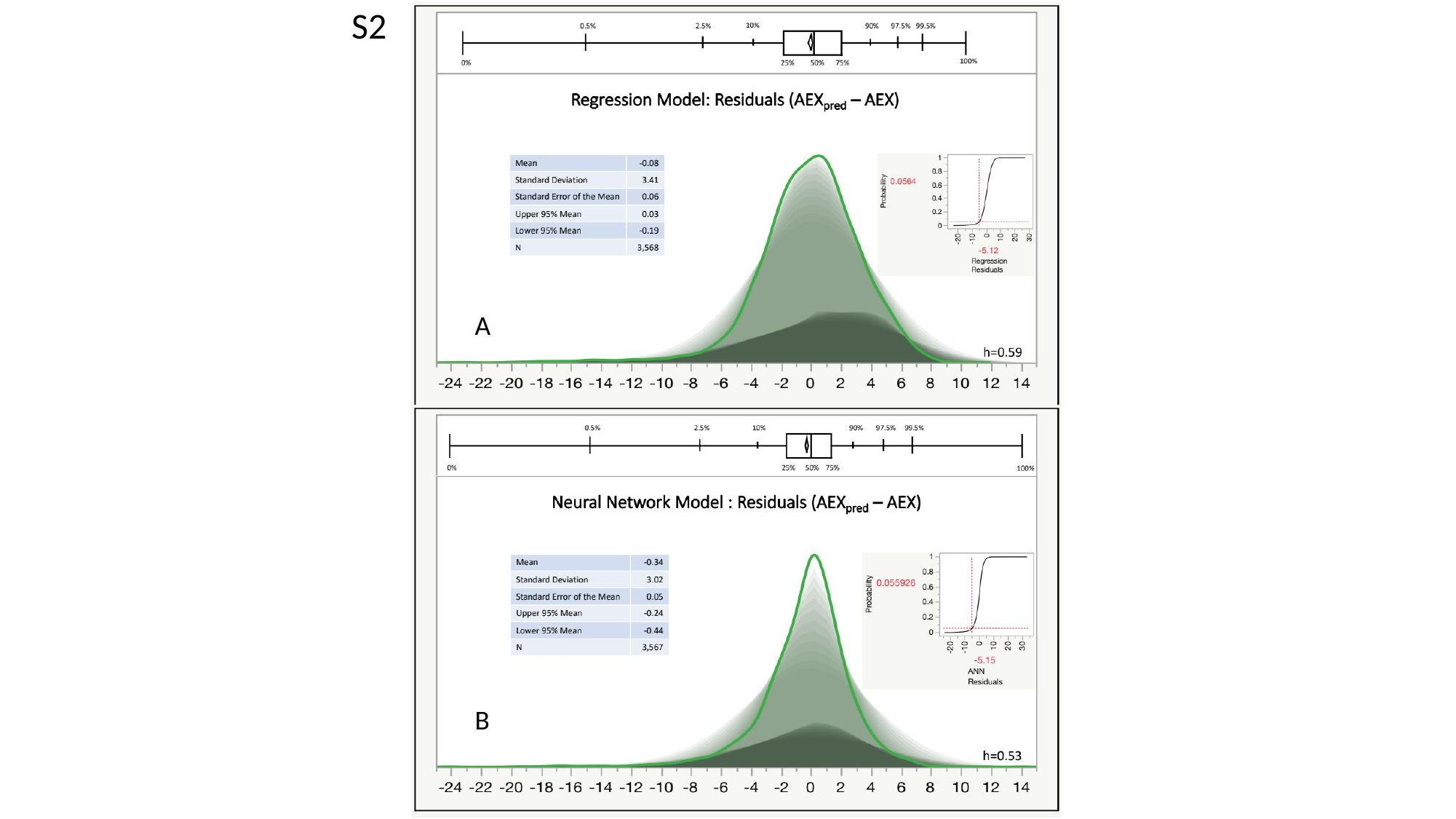

S2
A
B

Supplement: Supplementary file 3 — Supplementary Figure S2. [file 41598_2020_73925_MOESM3_ESM.pptx]
