# Supplementary material for: Area under the expiratory flow-volume curve: predicted values by artificial neural networks
Source: Sci Rep. 2020 Oct 6;10:16624. doi: 10.1038/s41598-020-73925-0 (PMC7538954; doi:10.1038/s41598-020-73925-0)
Supplement: Supplementary file 4 — Supplementary Figure S3. [file 41598_2020_73925_MOESM4_ESM.pptx]

## Slide 1
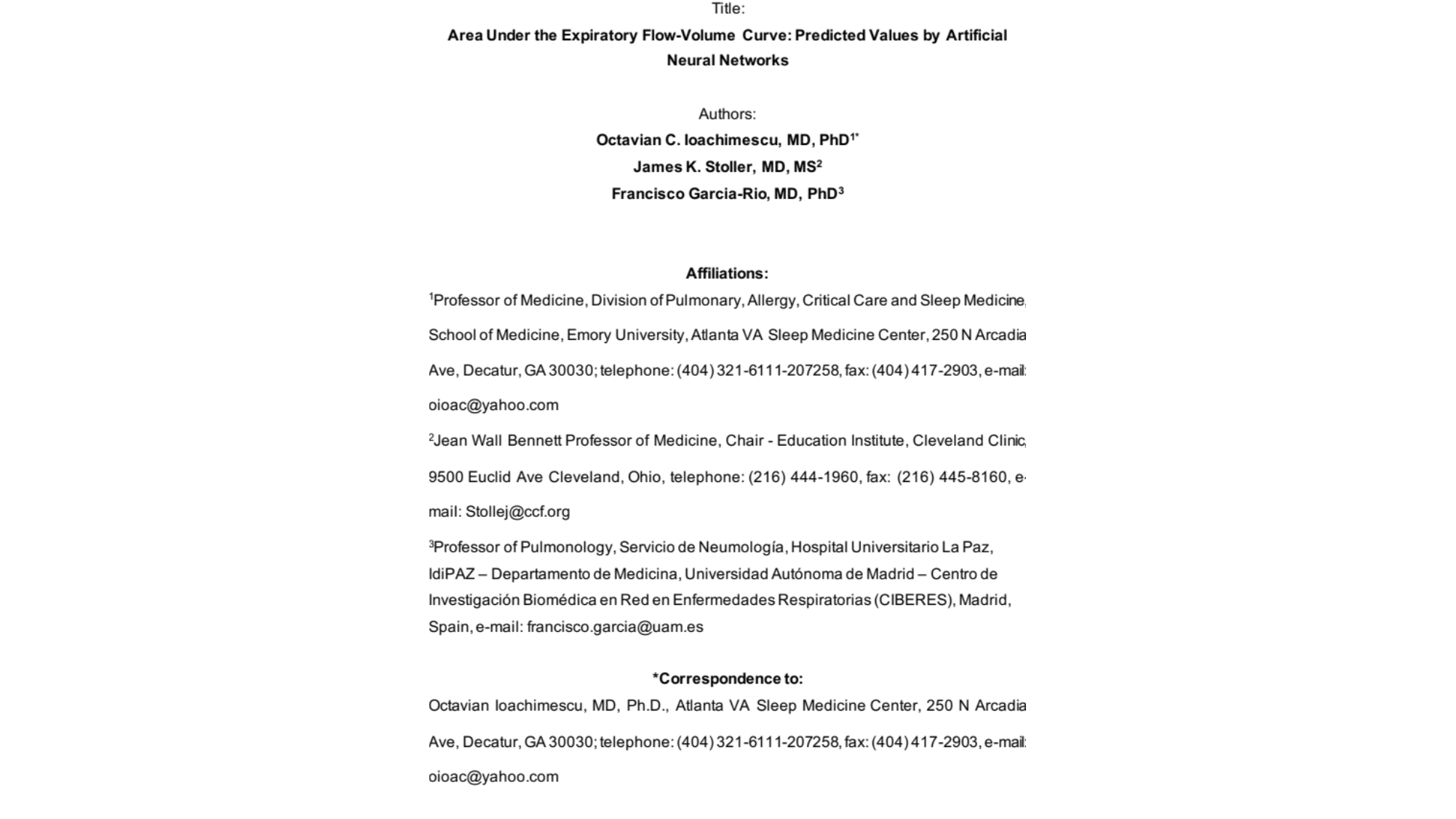

## Slide 2
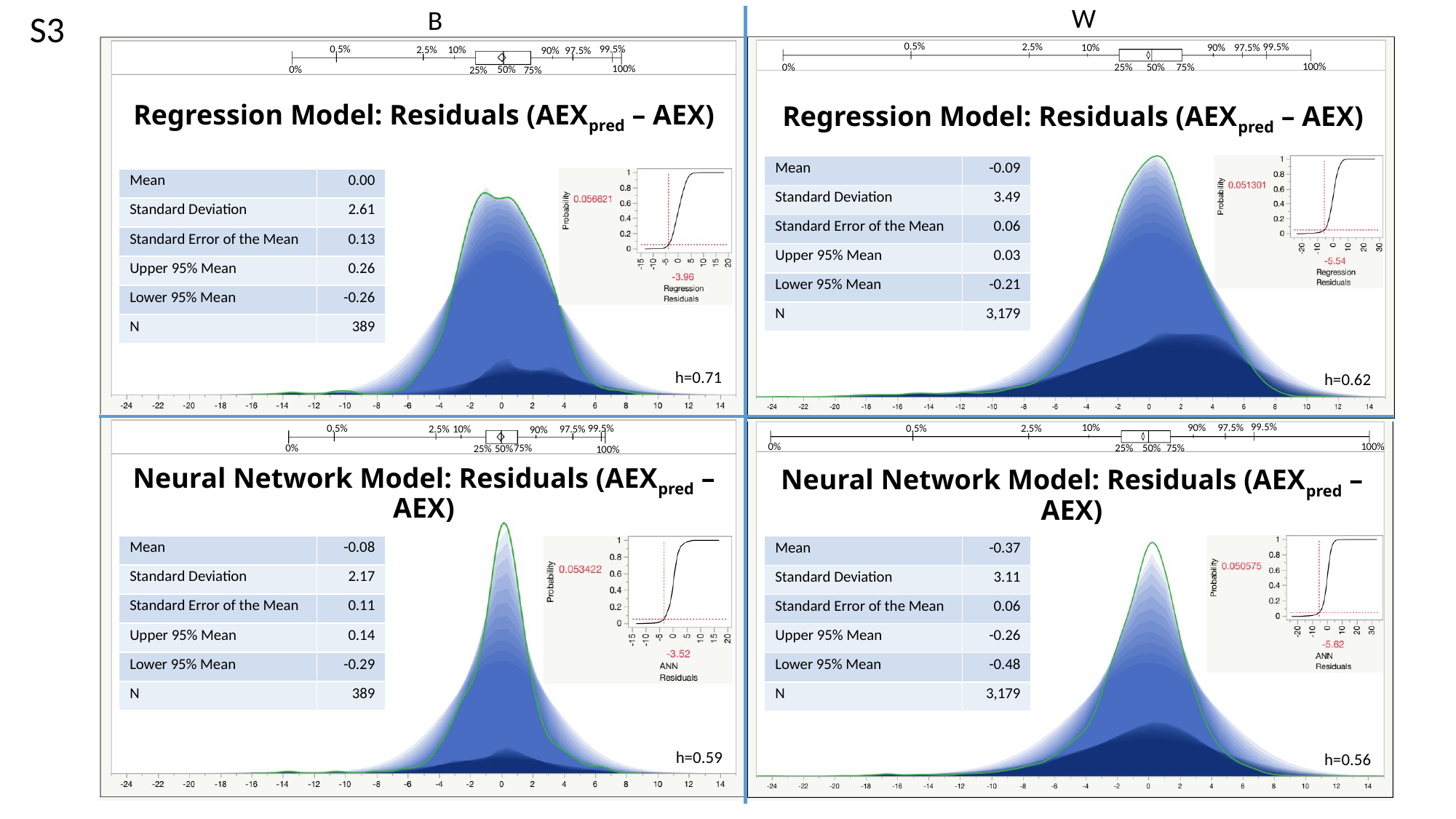

W
B
S3
0.5%
99.5%
2.5%
97.5%
90%
10%
0.5%
99.5%
10%
2.5%
90%
97.5%
100%
75%
0%
25%
50%
100%
0%
50%
25%
75%
# Regression Model: Residuals (AEXpred – AEX)
Regression Model: Residuals (AEXpred – AEX)
| Mean | -0.09 |
| --- | --- |
| Standard Deviation | 3.49 |
| Standard Error of the Mean | 0.06 |
| Upper 95% Mean | 0.03 |
| Lower 95% Mean | -0.21 |
| N | 3,179 |
| Mean | 0.00 |
| --- | --- |
| Standard Deviation | 2.61 |
| Standard Error of the Mean | 0.13 |
| Upper 95% Mean | 0.26 |
| Lower 95% Mean | -0.26 |
| N | 389 |
h=0.71
h=0.62
99.5%
90%
97.5%
10%
0.5%
2.5%
0.5%
99.5%
2.5%
97.5%
10%
90%
0%
100%
50%
75%
25%
0%
75%
50%
25%
100%
Neural Network Model: Residuals (AEXpred – AEX)
Neural Network Model: Residuals (AEXpred – AEX)
| Mean | -0.08 |
| --- | --- |
| Standard Deviation | 2.17 |
| Standard Error of the Mean | 0.11 |
| Upper 95% Mean | 0.14 |
| Lower 95% Mean | -0.29 |
| N | 389 |
| Mean | -0.37 |
| --- | --- |
| Standard Deviation | 3.11 |
| Standard Error of the Mean | 0.06 |
| Upper 95% Mean | -0.26 |
| Lower 95% Mean | -0.48 |
| N | 3,179 |
h=0.59
h=0.56
